# Supplementary material for: CuMV VLPs Containing the RBM from SARS-CoV-2 Spike Protein Drive Dendritic Cell Activation and Th1 Polarization
Source: Pharmaceutics. 2023 Mar 2;15(3):825. doi: 10.3390/pharmaceutics15030825 (PMC10055701; doi:10.3390/pharmaceutics15030825)
Supplement: Supplementary file 1 [file pharmaceutics-15-00825-s001.zip › pharmaceutics-2172345-supplementary.pdf]

Supplementary Material

Supplementary Table S1: Primer sequences for studied genes

Gene Symbol	Batch	5´-3´sequence<br>F: forward; R:reverse
TBP	HA12823724	F: GCCAAGAGTGAAGAACAG
	HA12823725	R: GAAGTCCAAGAACTTAGCTG
IL6	HA14200417	F: GCAGAAAAAGGCAAAGAATC
	HA14200418	R: CTACATTTGCCGAAGAGC
IL10	HA14200419	F: GCCTTTAATAAGCTCCAAGAG
	HA14200420	R: ATCTTCATTGTCATGTAGGC
IL15	HA14200381	F: AGCAATGTTCCATCATGTTC
	HA14200382	R: ATACGATCTTGTATGGGCTG
IL18	HA14200383	F: CCTTTAAGGAAATGAATCCTCC
	HA14200384	R: CATCTTATTATCATGTCCTGGG
IL12A	HA14200377	F: AAGACCTCTTTTATGATGGC
	HA14200378	R: CATTCATGGTCTTGAACTCC
IL12B	HA14200379	F: AGAAAAGATAGAGTCTTCACGG
	HA14200380	R: AAGATGAGCTATAGTAGCGG
IL1β	HA14200415	F: CTAAACAGATGAAGTGCTCC

	HA14200416	R: GGTCATTCTCCTGGAAGG
CXCR4	HA14200409	F: AACTTCAGTTTGTTGGCTG
	HA14200410	R: GTGTATATACTGATCCCCTCC
CCR7	HA14200399	F: TTGTCATTTTCCAGGTATGC
	HA14200400	R: AATGATGGAGTACATGATAGGG
CCL22	HA14200425	F: GTGGTGTTGCTAACCTTC
	HA14200426	R: GGCTCAGCTTATTGAGAATC
TGFβ	HA14200407	F: AACCCACAACGAAATCTATG
	HA14200408	R: CTTTTAACTTGAGCCTCAGC
TNFα	HA14200429	F: AGGCAGTCAGATCATCTTC
	HA14200430	R: TTATCTCTCAGCTCCACG
iNOS	HA14364086	F: CATCAACCAGTATTATGGCTC
	HA14364087	R: TTCCTTTGTTACAGCTTCC
